# Development of a risk prediction nomogram for sarcopenia in hemodialysis patients

**DOI:** 10.1186/s12882-022-02942-0

**Published:** 2022-09-23

**Authors:** Genlian Cai, Jinping Ying, Mengyan Pan, Xiabing lang, Weiping Yu, Qinqin Zhang

**Affiliations:** Kidney Disease Center, The First Affiliated Hospital, Zhejiang University School of Medicine, #1367 Wenyixi Road, Hangzhou, 311121 China

**Keywords:** Sarcopenia, Hemodialysis, Nomogram

## Abstract

**Background:**

Sarcopenia is associated with various adverse outcomes in hemodialysis patients. However, current tools for assessing and diagnosing sarcopenia have limited applicability. In this study, we aimed to develop a simple and reliable nomogram to predict the risk of sarcopenia in hemodialysis patients that could assist physicians identify high-risk patients early.

**Methods:**

A total of 615 patients undergoing hemodialysis at the First Affiliated Hospital College of Medicine Zhejiang University between March to June 2021 were included. They were randomly divided into either the development cohort (*n* = 369) or the validation cohort (*n* = 246). Multivariable logistic regression analysis was used to screen statistically significant variables for constructing the risk prediction nomogram for Sarcopenia. The line plots were drawn to evaluate the effectiveness of the nomogram in three aspects, namely differentiation, calibration, and clinical net benefit, and were further validated by the Bootstrap method.

**Results:**

The study finally included five clinical factors to construct the nomogram, including age, C-reactive protein, serum phosphorus, body mass index, and mid-upper arm muscle circumference, and constructed a nomogram. The area under the ROC curve of the line chart model was 0.869, with a sensitivity and specificity of 77% sensitivity and 83%, the Youden index was 0.60, and the internal verification C-statistic was 0.783.

**Conclusions:**

This study developed and validated a nomogram model to predict the risk of sarcopenia in hemodialysis patients, which can be used for early identification and timely intervention in high-risk groups.

## Introduction

Hemodialysis (HD) is one of the most common treatments for patients with end-stage renal disease (ESRD) [1]. Epidemiology studies [2] report that approximately 84% of all ESRD patients eventually receive hemodialysis treatment. Previous studies have indicated that patients with HD are predisposed to sarcopenia owing to the chronic inflammatory status, metabolic acidosis, malnutrition, and decreased physical activity [3]. Sarcopenia is characterized by a progressive and systemic loss of muscle mass and strength/function that is typically associated with numerous adverse outcomes [4, 5]. It has been estimated that 20–50% of all HD patients will develop sarcopenia, which is much higher than the general population [6–9]. Earlier studies have revealed that HD patients with comorbid sarcopenia had an increased risk of falls, fractures, and cardiovascular events as well as a higher likelihood re-hospitalization and death [10–12]. Two recent systematic review and meta-analysis have also corroborated that sarcopenia is associated with a higher risk of death in hemodialysis patients [13, 14]. Other studies have demonstrated that that early identification and timely intervention can lower the occurrence and development of sarcopenia in hemodialysis patients [15, 16]. Therefore, the importance of early identification of high-risk groups of sarcopenia in hemodialysis patients cannot be overstated.

The diagnosis of sarcopenia is mainly based on low skeletal muscle mass, skeletal muscle strength, and physical performance [4]. Several technologies are currently being employed to estimate skeletal muscle mass, including magnetic resonance imaging (MRI), computed tomography (CT), dual-energy X-ray absorptiometry (DXA) and bioelectrical impedance analysis (BIA). Nevertheless, the use of DXA, MRI, and CT is limited in clinical practice by disadvantages such as high cost, complex procedures, and radiation exposure [17]. On the other hand, even though the BIA is convenient to perform, however, it was not applicable for the HD patients who experienced pacemaker implant and amputation [18]. The Asian Working Group for Sarcopenia (AWGS)2019 update proposes screening for sarcopenia with either SARC-F or SARC-CalF to facilitate earlier identification of high-risk individuals [15]. SARC-F is a self-reported questionnaire based on the patient’s perception of limitations in strength, walking ability, rising from a chair, stair climbing, and falls. The SARC-F score ≥ 4 indicates a risk of sarcopenia, however, numerous studies have demonstrated the low sensitivity and high specificity of SARC-F [19, 20]. SARC-CalF enhanced the sensitivity of SARC-F by the inclusion of calf circumference, with a score ≥ 11 indicates a risk of sarcopenia [21]. Despite the fact that SARC-F or SAC-CalF is simple to use and cost-free for identifying the risk of sarcopenia in hemodialysis patients, their results have been controversial [22, 23]. The relatively low sensitivity of these two scales renders a higher risk for miss diagnosis. As a result, the clinical medical staff cannot promptly identify the high-risk groups of sarcopenia in hemodialysis patients and thereby miss the window for early intervention.

To address the aforementioned challenges, we aimed to develop and validate a reliable nomogram model for predicting sarcopenia risk in hemodialysis patients. With this model, we hope to early identification of hemodialysis patients at high risk for sarcopenia and enable timely interventions to prevent or slow development and progression of sarcopenia. Thus improving patients adverse outcomes.

## Methods

### Study population

All patients undergoing hemodialysis at the First Affiliated Hospital College of Medicine Zhejiang University from March to June 2021 were enrolled in our study, and their medical records were retrospectively reviewed. Patients aged ≥ 18 years with a history of hemodialysis three times or more per week for at least 3 months were eligible to participate in this study. The exclusion criteria included implanted pacemaker or amputation surgeries; miss data of skeletal muscle mass index. Finally, 615 patients were enrolled in our study. They were randomly allocated to either the development cohort (*n* = 369) or the validation cohort (*n* = 246) in a 6:4 ratio. This study was performed in accordance with Declaration of Helsinki and was approved by the Clinical Research Ethics Committee of the First Affiliated Hospital of Zhejiang University School of Medicine (IIT20210808A), and all patients signed the informed consent form.

### Data collection

Based on the relevant literature [3, 6, 8, 12, 24] and consulting experts, a total of 27 predictors for sarcopenia were identified. The following data were collected from each patient: ① baseline demographics: age, gender, primary disease, duration of dialysis, etc.; ② self-rating anxiety scale (SAS) and self-rating depression scale (SDS) psychological status assessment indicators; ③ anthropometric indicators: height, post-dialysis weight, skeletal muscle mass index (SMI), handgrip strength, gait speed, mid-upper arm circumference, triceps skinfold thickness, body mass index (BMI) and mid-upper arm muscle circumference (MAMC),which was calculated as mid-upper arm circumference − 3.14 × triceps skinfold thickness [18]. ④ laboratory examination parameters measured before a mid-week predialysis session following enrolment: serum creatinine, serum uric acid, serum urea nitrogen, hemoglobin, serum albumin, serum prealbumin, C-reactive protein, serum phosphorus, serum calcium, blood lipid, serum potassium, parathyroid hormone and urea clearance index.

Handgrip strength test was measured before hemodialysis with an electronic grip strength meter (EH101; CAMRY). The patient was required to stand the feet and arms naturally positioned. Three measurements with an interval of 5 s were taken, and the maximum value was recorded. The gait speed test was also evaluated before hemodialysis, without the assistance of any tools, the patient walked 6 m twice at a normal gait speed, each gait speed was observed, and the mean value of two consecutive measurements was documented. Triceps skinfold thickness and upper arm circumference were measured at the end of hemodialysis. In addition, BIA (InbodyS10, BiospaceCo,Korea) was performed 15–20 min after hemodialysis to measure appendicular skeletal muscle mass (ASM), which was later used to calculate SMI as follows: SMI = ASM/height^2^ [15].

The Zung self-rating depression scale (SDS) and Zung self-rating anxiety scale (SAS) were employed to assess patients' depression and anxiety levels; SAS and SDS have been found to have robust internal consistency with a Cronbach s alpha of 0.82 and 0.68, respectively. Both the SAS and SDS are measured on a scale containing 20 items, with each item scored on a four-point Likert scale, with “1” indicating no or little time, “2” representing a small amount of time, “3” referring to a lot of time, and “4” representing most or all of the time. The raw scores range from 20 to 80, which are subsequently converted into standard scores by dividing the sum of the raw scores by 80 and multiplying by 100 for further evaluation. the thresholds for identifying depression and anxiety were 50 points [25, 26].

Demographic characteristics and laboratory examination parameters were collected from medical records. The anthropometric indicators and SAS and SDS scales were determined by experienced nursing researchers; SAS and SDS were explained to patients by researchers using standardized guiding terms and were then filled in by the patients.

### Diagnosis

In this study, the diagnosis of sarcopenia was based on the AWGS 2019 criteria [27]. ①low appendicular skeletal muscle mass (ASM): BIA < 7.0 kg/m^2^ in males and < 5.7 kg/m^2^ in females ②low muscle strength: handgrip strength < 28 kg in males and < 18 kg in females; ③low physical performance: gait speed (6-m walk): < 1.0 m/s. The diagnosis of sarcopenia was made in patients with low ASM + low muscle strength OR low physical performance.

### Statistical analysis

SPSS 26 software (IBM Corporation, Armonk, NY, USA) and R software (version 4.2.1, R Foundation for Statistical Computing, Vienna, Austria) were used to analyze the collected data. Among the 27 predictor variables, prealbumin, urea clearance index, parathyroid hormone, upper arm circumference, and skinfold thickness were identified with less than 5% missing data, which were replaced by the series mean of the continuous variables before the model was developed. To ensure more convenient clinical use, continuous variables such as age, serum albumin, serum creatinine, C-reactive protein, serum phosphorus, serum calcium, parathyroid hormone, triglycerides, cholesterol, high-density lipoprotein, low-density lipoprotein, and very-low-density lipoprotein, BMI were converted into categorical variables according to clinical references [27–30]. Other continuous variables were converted to dichotomous variables according to the receiver operating characteristic curve using the optimal cut-off points of the analyzed variables with the Youden index criterion. Frequency or constituent ratio was used for statistical description, and the chi-square test was used for comparison between groups. Through univariate logistic regression analysis, variables with univariate analysis results of *p* < 0.05 were included in the multivariate logistic regression analysis. The backward stepwise regression method was then applied to select statistically significant factors used to construct the clinical risk prediction nomogram. The predictive nomogram was assessed in three aspects: discrimination, calibration, and clinical net benefit. The area under the receiver operating characteristic curve (ROC) was utilized to assess discriminatory ability. The calibration curve and Hosmer–Lemeshow goodness of fit test were used to assess calibration ability. The decision curve analysis (DCA) was used to assess clinical effectiveness. The model was internally validated using the Bootstrap method(resampling = 1000). A *p*-value < 0.05 was considered statistically significant.

## Results

### Demographic parameters

**Fig. 1:**
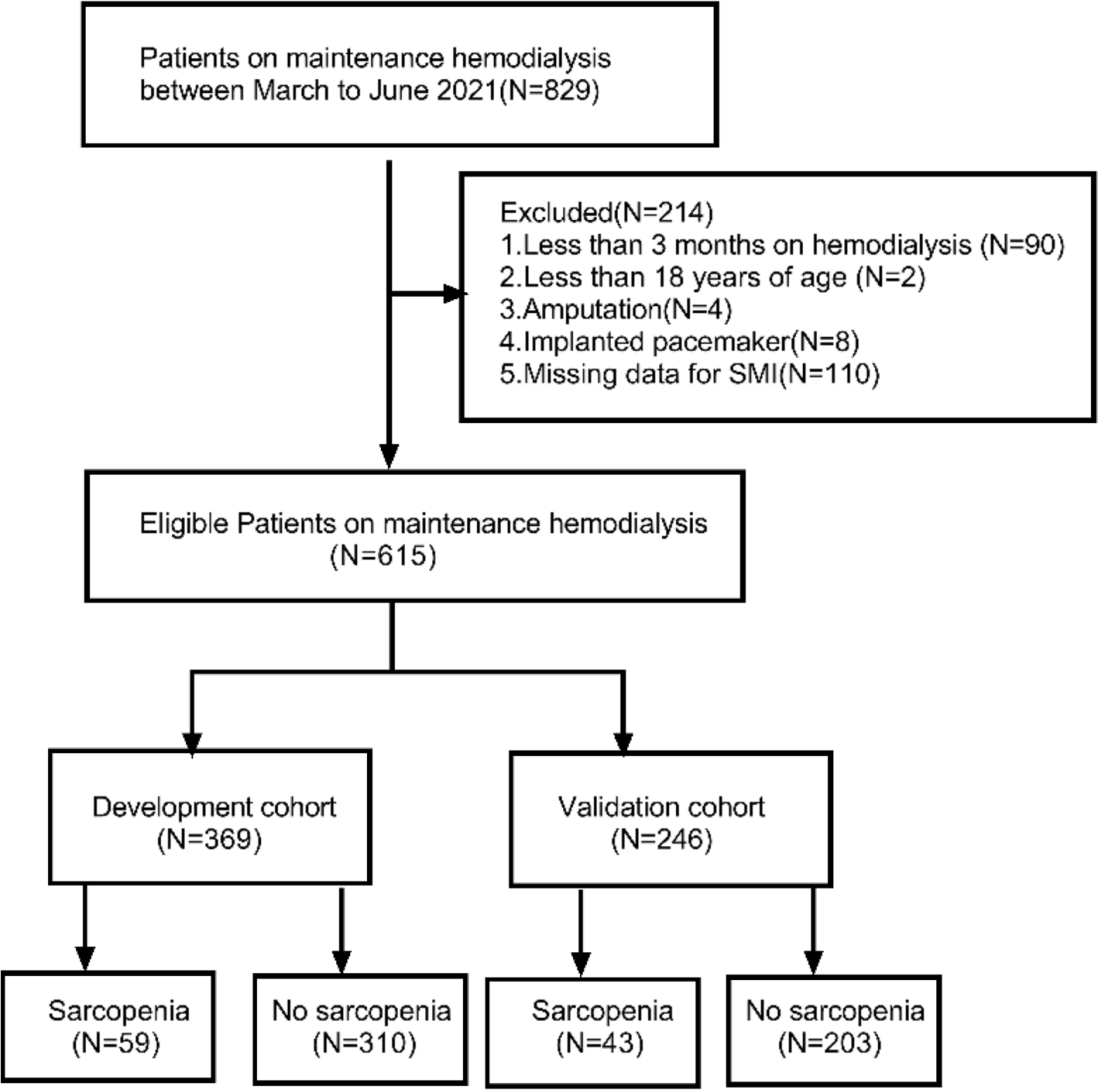
A workflow to develop the prediction models for sarcopenia in HD patients

A total of 615 patients undergoing hemodialysis were eventually included in this study and were randomly divided into the development cohort (*n* = 369) and the validation cohort (*n* = 246) in a 6:4 ratio, as illustrated in (Fig. 1). Among the enrolled patients, 381 (62%) were male and 234 (38%) were female. The mean patient age was 60.07 ± 14.34 years, the median duration of dialysis was 60 months (interquartile range 21–110). The most common reason for dialysis was chronic glomerulonephritis (430 patients), followed by diabetic nephropathy (101 patients), and other causes (hypertensive nephropathy, polycystic kidney disease, lupus nephritis, etc.). There were 102 patients (16.60%) diagnosed with sarcopenia and the demographic parameters of patients with or without sarcopenia are summarized in (Table 1).

**Table 1:** Baseline characteristics of the development and validation cohorts (n,%)

Characteristics	Development cohort(*N* = 369)	Validation cohort(*N* = 246)	*P* value
**Sociodemographic**			
Gender			
Female	138(37.40)	96(39.00)	0.684
Male	231(62.60)	150(61.00)	
Age (≥ 60 years)	176(47.70)	126(51.20)	0.392
**Sarcopenia**			
No	310(84.00)	203(82.50)	0.626
Yes	59(16.00)	43(17.50)	
**Dialysis and clinical**			
Primary disease			
Chronic glomerulonephrit	259(70.20)	171(69.50)	0.110
Diabetic nephropathy	67(18.20)	34(13.80)	
Other	43(11.70)	41(16.70)	
Duration of dialysis (> 60 months)	179(48.50)	128(52.00)	0.392
**Biochemical**			
Hemoglobin (< 110 g/L)	149(40.40)	97(39.40)	0.814
Creatinine (< 884 μmol/L)	175(47.40)	108(43.90)	0.390
Uric acid (> 420 μmol/L)	201(54.50)	147(59.80)	0.195
Urea nitrogen (> 20 mmol/L)	107(29.00)	69(28.00)	0.799
Kt/V (> 1.4)	248(67.20)	168(68.30)	0.778
Serum Albumin(< 38 g/L)	107(29.00)	85(34.60)	0.145
CRP (≥ 3 mg/L)	140(37.90)	101(41.10)	0.438
Alkaline phosphatase (> 70.5U/L)	194(52.60)	137(55.70)	0.448
Prealbumin (≤ 30 mg/dL)	140(37.90)	85(34.60)	0.393
Ferritin (< 200 ng/mL)	251(68.00)	171(69.50)	0.696
Potassium (> 5.5 mmol/L)	104(28.20)	51(20.70)	0.037
Phosphorus			
1.13–1.78 mmol/L	148(40.10)	91(36.99)	0.707
> 1.78 mmol/L	208(56.40)	147(59.76)	
< 1.13 mmol/L	13(3.50)	8(3.25)	
Calcium			
2.1–2.5 mmol/L	243(65.86)	146(59.30)	0.149
> 2.5 mmol/L	63(17.07)	43(17.50)	
< 2.1 mmol/L	63(17.07)	57(23.20)	
PTH			
150-300 pg/L	138(37.40)	84(34.14)	0.180
> 300 pg/L	74(20.10)	65(26.43)	
< 150 pg/L	157(42.50)	97(39.43)	
Triglycerides (> 1.7 mmol/L)	175(47.40)	119(48.40)	0.818
Cholesterol (< 2.59 mmol/L)	41(11.10)	36(14.60)	0.196
HDL (< 1.03 mmol/L)	141(38.20)	93(37.80)	0.919
LDL (> 3.37 mmol/L)	7(1.90)	7(2.80)	0.440
VLDL (> 0.78 mmol/L)	171(46.30)	115(46.70)	0.921
**Anthropometric indicators**			
MAMC (< 22.64 cm)	151(40.90)	112(45.50)	0.258
Body mass index			0.978
18.5–24.9 kg/m2	243(65.85)	164(66.67)	
> 25 kg/m2	55(14.91)	36(14.63)	
< 18.5 kg/m2	71(19.24)	46(18.70)	
**Psychological scores**			
SAS (> 40)	23(6.20)	13(5.30)	0.624
SDS (> 41)	41(11.10)	33(13.40)	0.390

*Kt/V* Urea clearance index, *CRP* C-reactive protein, *MAMC* Mid-upper arm muscle circumference, *SAS* Self-rating anxiety scale, *SDS* Self-rating depression scale, *PTH* Parathyroid hormone, *HDL* High-density lipoprotein, *LDL* Low-density lipoprotein, *VLDL* Very low-density lipoprotein

### Construction of the predictive nomogram

Univariate logistic regression analysis was performed with the included 27 independent variables, and the results demonstrated that age, urea clearance index, serum creatinine, upper arm muscle circumference, as well as levels of blood uric acid, albumin, C-reactive protein, serum phosphorus, alkaline phosphatase, BMI, prealbumin, and triglycerides were significantly different between the two groups (*P* < 0.05), as presented in (Table 2). Next, significant independent variables obtained from the above univariate logistic regression analysis were included in multivariate logistic regression analysis following a backward stepwise regression method. The results exposed that age, C-reactive protein, and serum phosphorus levels, as well as BMI and MAMC, were independent risk factors for sarcopenia in HD patients, as outlined in Table 2.

**Table 2:** Univariate and multivariate analysis of Sarcopenia in the Development cohort (*n* = 369)

	Univariate analysis				Multivariate analysis			
Characteristics	β	OR	95%CI	P	β	OR	95%	P
Gender (Female)	0.006	1.01	0.57–1.79	0.985				
Age(≥ 60 years)	0.901	2.46	1.37–4.42	0.002	0.651	1.92	0.94–3.93	0.075
Diabetic nephropathy	0.58	1.79	0.91–3.51	0.091				
Other	0.348	1.42	0.61–3.3	0.42				
Duration of dialysis (> 60 months)	0.273	1.31	0.75–2.3	0.338				
Hemoglobin (< 110 g/L)	0.344	1.41	0.81–2.47	0.228				
Creatinine (< 884 μmol/L)	1.388	4.01	2.14–7.5	< 0.001				
Uric acid (> 420 μmol/L)	-0.58	0.56	0.32–0.98	0.043				
Urea nitrogen (> 20 mmol/L)	0.542	1.72	0.97–3.07	0.067				
KTV (> 1.4)	1.297	3.66	1.68–7.98	0.001				
Serum Albumin (< 38 g/L)	0.886	2.43	1.37–4.3	0.002				
Alkaline phosphatase (> 70.5U/L)	0.58	1.79	1–3.18	0.049				
CRP (≥ 3 mg/L)	0.962	2.62	1.49–4.61	0.001	1.168	3.22	1.58–6.54	0.001
Prealbumin (≤ 30 mg/dL)	0.714	2.04	1.16–3.58	0.013				
Ferritin (< 200 ng/mL)	-0.369	0.69	0.39–1.23	0.21				
Potassium (> 5.5 mmol/L)	-0.167	0.85	0.45–1.6	0.607				
Phosphorus (> 1.78 mmol/L)	-0.871	0.42	0.23–0.77	0.005	-1.011	0.36	0.18–0.74	0.006
Phosphorus (< 1.13 mmol/L)	2.18	8.85	2.55–30.72	0.001	2.769	15.94	3.28–77.35	0.001
Calcium (> 2.5 mmol/L)	-0.304	0.74	0.33–1.67	0.466				
Calcium (< 2.1 mmol/L)	0.071	1.07	0.52–2.23	0.85				
PTH (> 300 pg/L)	-0.697	0.5	0.21–1.16	0.106				
PTH (< 150 pg/L)	-0.299	0.74	0.4–1.36	0.333				
Triglycerides (> 1.7 mmol/L)	-0.668	0.51	0.29–0.92	0.025				
Cholesterol (< 2.59 mmol/L)	-0.35	0.7	0.26–1.88	0.484				
HDL (< 1.03 mmol/L)	-0.536	0.59	0.33–1.03	0.061				
LDL (> 3.37 mmol/L)	-0.135	0.87	0.1–7.38	0.901				
VLDL (> 0.78 mmol/L)	-0.358	0.7	0.4–1.23	0.218				
MAMC (< 22.64 cm)	1.933	6.91	3.58–13.35	< 0.001	1.422	4.15	1.82–9.05	0.001
BMI (> 25 kg/m^2^)	-1.99	0.14	0.02–1.02	0.053	-2.122	0.12	0.01–1.14	0.065
BMI (< 18.5 kg/m^2^)	1.628	5.1	2.76–9.39	< 0.001	1.428	4.15	1.91–6.21	< 0.001
SAS (> 40)	0.668	1.95	0.74–5.18	0.179				
SDS (> 41)	0.447	1.56	0.7–3.47	0.272				

*Kt/V* Urea clearance index, *CRP C-reactive protein, MAMC* Mid-upper arm muscle circumference, *SAS* Self-rating anxiety scale, *SDS* Self-rating depression scale, *PTH* Parathyroid hormone, *HDL* High-density lipoprotein, *LDL* Low-density lipoprotein, *VLDL* Very low-density lipoprotein

**Fig. 2:**
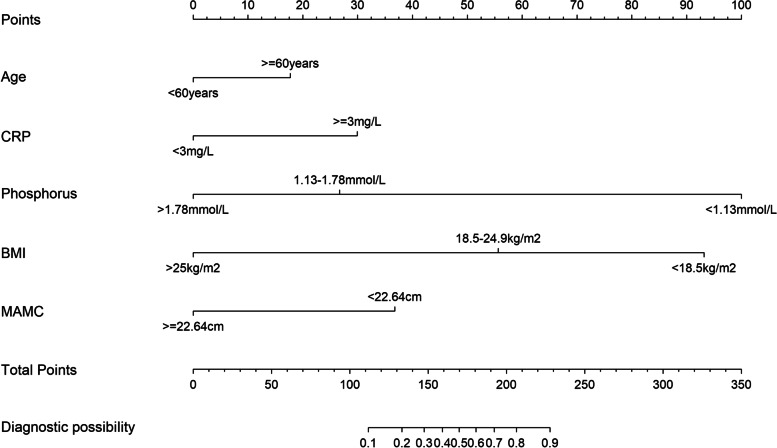
Nomogram to predicting the risk of sarcopenia in hemodialysis patients

The risk prediction model for sarcopenia in HD patients was then established based on the formula: *P* = 1/(1 + e^Y^) where e stands for the base of natural logarithm, Y = -3.316 + 0.651 × age ≥ 60 years + 1.168 × C-reactive protein ≥ 3 mg/L + 2.769 × serum phosphorus < 1.13 mmol/L-1.011 × serum phosphorus 1.13 ~ 1.78 mmol/L + 1.428 × BMI < 18.5 kg/m^2^ -2.122 × BMI 18.5 ~ 24.9 kg/m^2^ + 1.422 × MAMC < 22.64 cm. A nomogram of the risk prediction model for sarcopenia in HD patients was also plotted, as depicted in (Fig. 2). The corresponding score for each variable was obtained by crossing to the nomogram, and the total score was used to predict the risk of developing sarcopenia in HD patients.

### Validation of the predictive model

**Fig. 3:**
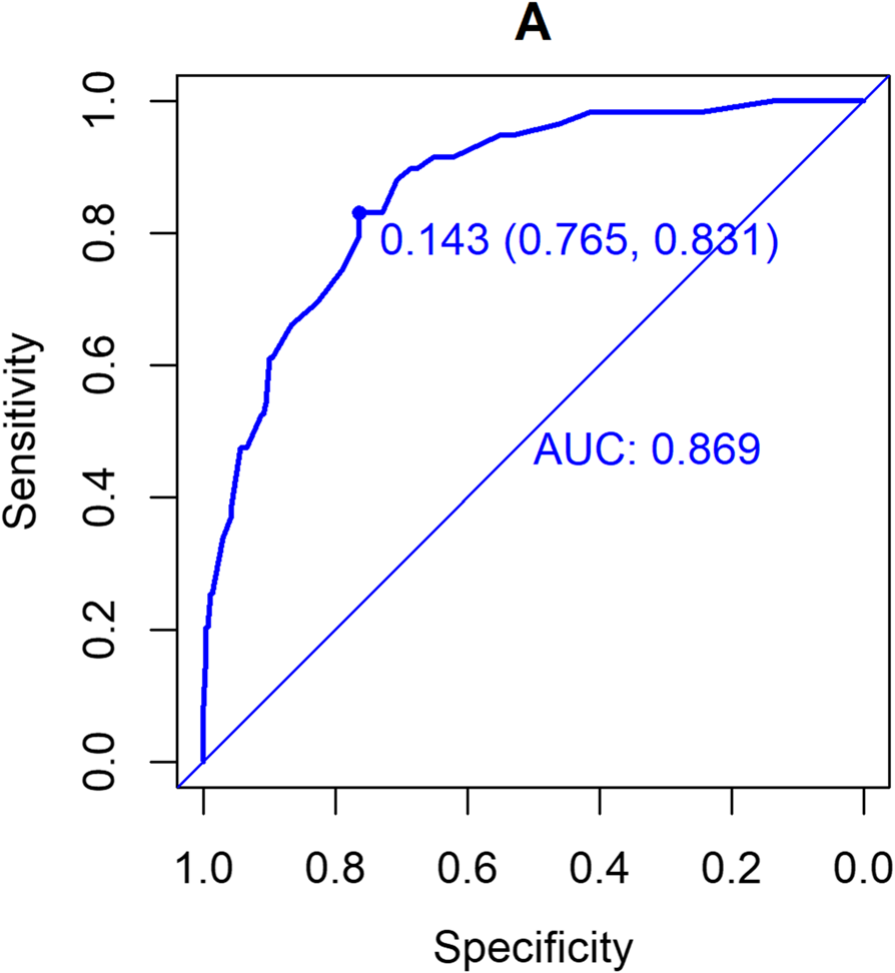
AUC of development cohort

**Fig. 4:**
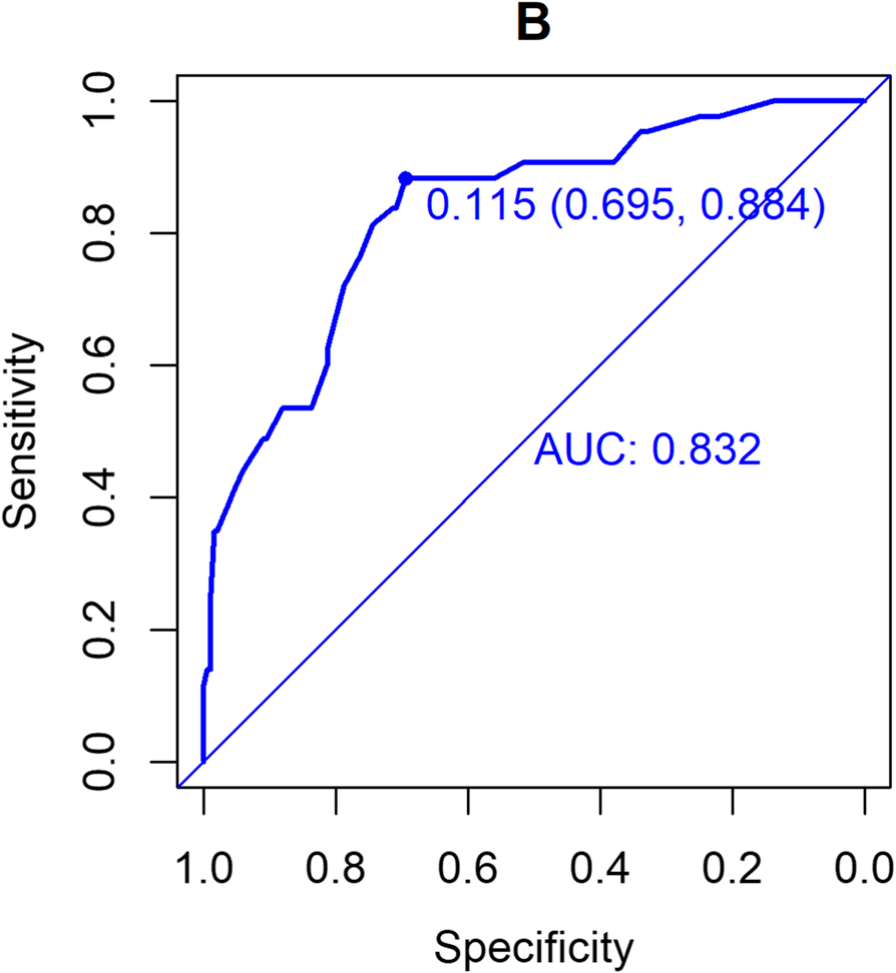
AUC of validation cohort

**Fig. 5:**
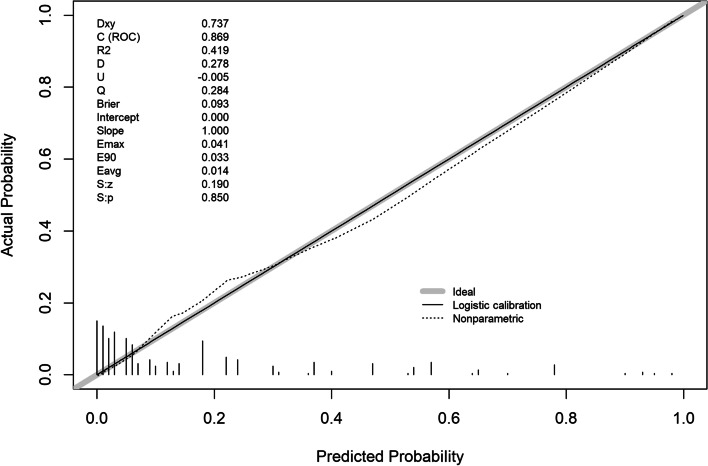
Calibration plot of development cohort

**Fig. 6:**
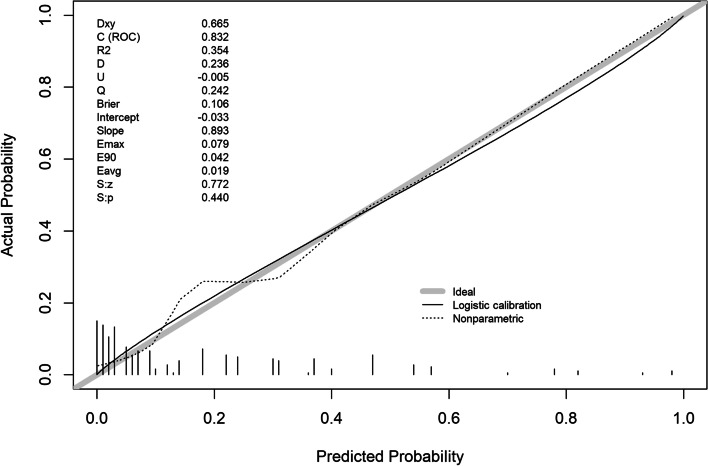
Calibration plot of validation cohort

The validation of this prediction model was performed by area under the curve and the calibration curve. Our data indicated that the AUC for this model was 0.869 (95% CI (0.822 to 0.915) (Fig. 3), with a sensitivity, specificity, and Youden index of 77% and 83%, and 0.60, respectively, in the development cohort. Similarly, the AUC of the validation cohort was 0.832 (95% CI (0.765 to 0.900) (Fig. 4), with a sensitivity of 70%, a specificity of 88%, and a Youden index of 0.58. In addition, the Hosmer–Lemeshow goodness-of-fit test showed promising fit (development cohort $\chi$ ^2^ = 4.001, *P* = 0.911; validation cohort $\chi$ ^2^ = 13.941, *P* = 0.124). In the development cohort and validation cohort, the calibration curve showed agreement between the observation and prediction as displayed in (Figs. 5 and 6). The model was then internally validated using the Bootstrap method with an internal validation C-statistic of 0.783.

### Clinical validity

**Fig. 7:**
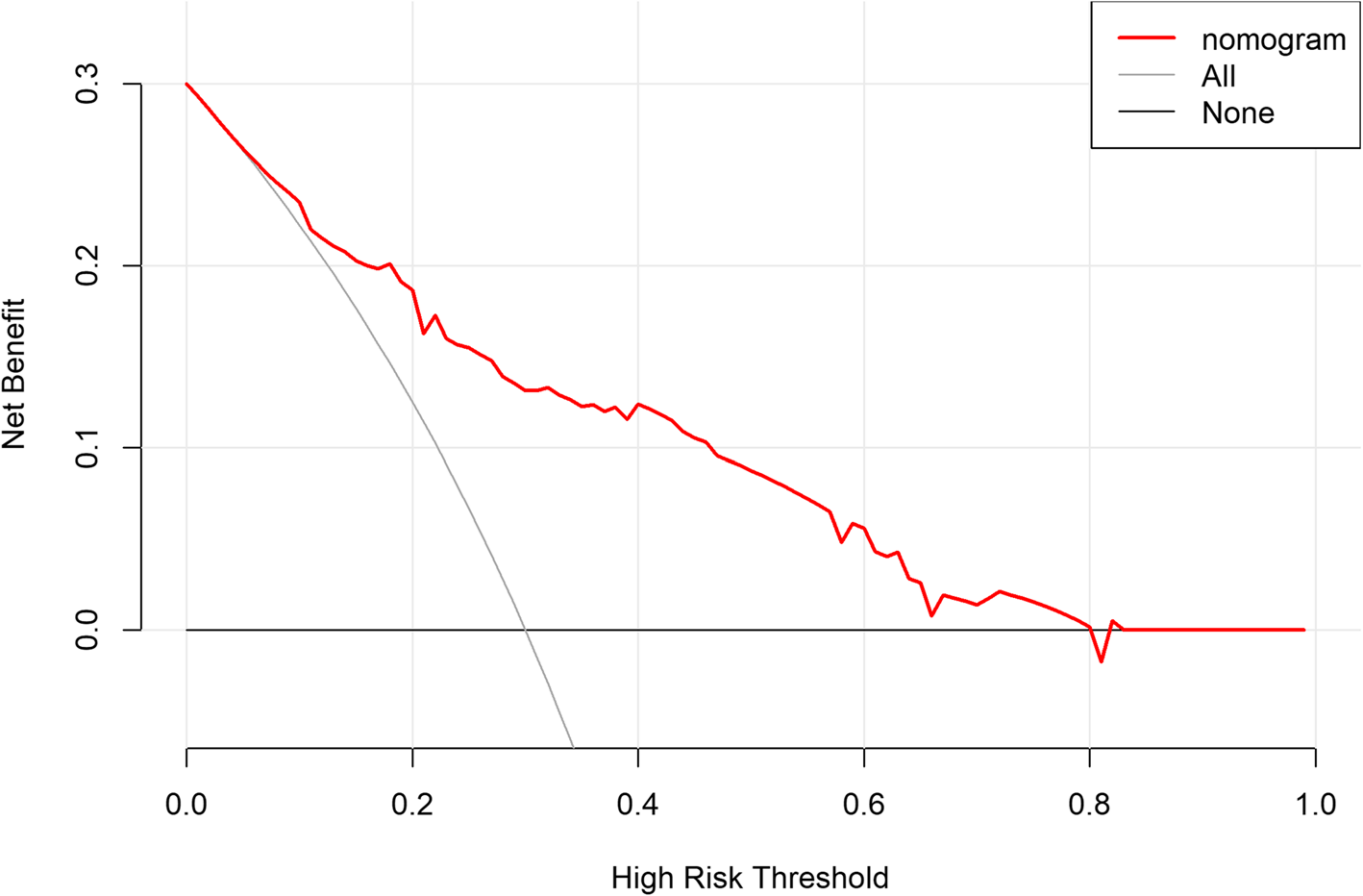
Decision curve analysis for development cohort

**Fig. 8:**
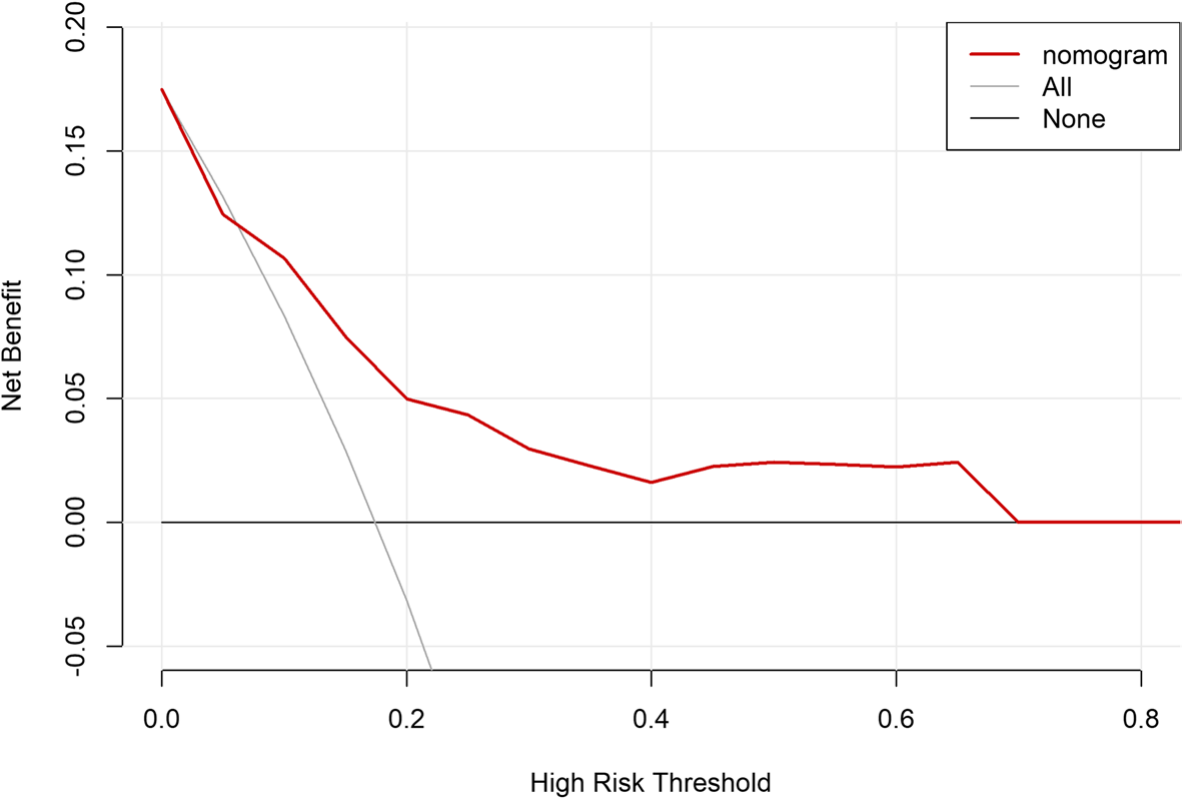
Decision curve analysis for validation cohort

The clinical validity of this predictive model was assessed using decision curve analysis (DCA). The DCA for the development and validation cohorts are displayed in (Figs. 7 and 8), which signaled that patients could benefit from this novel predictive model when the threshold was set to 10 ~ 80% and 10 ~ 70% for the development and validation cohorts, respectively. The positive predictive value, negative predictive value, positive likelihood ratio, negative likelihood ratio for this nomogram were 45%,93%,4.34,0.418, Accuracy was 82%.

To make this predictive model more convenient for physicians to use in clinical practice, we modified the nomogram into a scoring system with integer points: age ≥ 60 years(18points), C-reactive protein ≥ 3 mg/L (30points), serum phosphorus < 1.13 mmol/L (100points), serum phosphorus 1.13 ~ 1.78 mmol/L (27 points) and BMI < 18.5 kg/m^2^ (93 points), BMI 18.5 ~ 24.9 kg/m^2^ (56 points), MAMC < 22.64 cm(37 points). Then, the risk score model of sarcopenia in hemodialysis patients was established. The total score was 0–350 points of the nomogram, and the higher of the total score, the higher the risk of sarcopenia. The weights for each feature are list in Table 3 for calculation without a nomogram. the AUC of the scoring system was 0.867 (95% CI (0.830 to 0.903), with a sensitivity, specificity, and Youden index of 88% and 73%, and 0.611. The optimal cutoff value of 121 was taken as the risk threshold, if the total score < 121 points was classified as the low-risk group of sarcopenia, and the total score > 121 points was classified as the high-risk group.

**Table 3:** The points for predictors

Predictor	points
**Age**	
< 60 years	0
≥ 60 years	18
**CRP**	
< 3 mg/L	0
≥ 3 mg/L	30
**Serum Phosphorus**	
1.13–1.78 mmol/L	27
> 1.78 mmol/L	0
< 1.13 mmol/L	100
**BMI**	
18.5–24.9 kg/m2	56
≥ 25 kg/m2	0
< 18.5 kg/m2	93
**MAMC**	
≥ 22.64 cm	0
< 22.64 cm	37
**Total points**	**Risk of sarcopenia**
112	0.1
133	0.2
147	0.3
159	0.4
170	0.5
181	0.6
192	0.7
206	0.8
228	0.9

*CRP* C-reactive protein, *BMI* Body mass index, *MAMC* Mid-upper arm muscle circumference

## Discussion

Herein, we developed and validated a simple nomogram to predict the risk of developing sarcopenia in HD patients with a total of five clinically relevant variables, including age, C-reactive protein, serum phosphorus, BMI, and MAMC. The AUC, internal validation C-statistic, calibration curve, and DCA curve were constructed to validate the reliability as well as the accuracy of this model. The nomogram model can early identify hemodialysis patients at risk of sarcopenia, and enable timely interventions to prevent or slow development and progression. thus improving patients adverse outcomes.

Nomograms can predict the probability of disease by analyzing and integrating identified disease risk factors, thus providing valuable information for better clinical decisions. It has been extensively used in oncology and chronic diseases worldwide [24]. For instance, Cheng et al. [31] designed a nomogram to predict the risk of initiating renal replacement therapy within 3 years in diabetic nephropathy patients, while Jing et al. [32] developed a nomogram comprising multiple echocardiographic measures to assess 3-year all-cause mortality in hemodialysis patients, both of which showed favorable accuracy and reliability. Ouyang et al. [33]developed and validated an easy-to-use nomogram that can accurately predict 1-year, 5-year, and 10-year survival in hemodialysis patients. In addition, Mo, et al. [34] reported the development of nomograms to predict sarcopenia in community older adults. However, to the best of our knowledge, no study has been conducted to construct a nomogram that can predict the risk of developing sarcopenia in HD patients.

In this study, the multivariate logistic regression analysis indicated that age was a risk factor for sarcopenia in HD patients. Aging is a well-known independent risk factor for sarcopenia, With the increase of age, the protein breakdown and anabolic metabolism of patients are gradually unbalanced. In addition, mitochondrial dysfunction and hormonal changes caused by aging are also related to sarcopenia [5]. Our findings on the relationship between age and sarcopenia are consistent with previous studies [6, 8, 35]. However, *there* are some controversies, Wang et al. [14] systematic review have shown the age of HD patients was no significant influence on sarcopenia prevalence.

Hyperphosphatemia is a common complication in HD patients and is closely related to an increased risk of vascular calcification and cardiovascular mortality [36]. Interestingly, our study indicated that lower serum phosphorus level correlated with the development of sarcopenia in HD patients, which was in line with the findings of Ren et al. [8]. We hypothesized that a high-protein diet is the main source of phosphorus for uremic patients who often suffer from loss of appetite or anorexia. A decrease in food intake will inevitably lead to a decrease in serum phosphorus, malnutrition, and protein-energy expenditure in patients, ultimately resulting in sarcopenia [37]. Previous studies have shown that shown protein restriction to correlate with increased mortality in patients undergoing HD, restricting dietary protein to help control phosphorus levels in patients undergoing maintenance HD may be more harmful than beneficial [36]. Balancing Nutrition and Serum Phosphorus in HD patients requires an individualized approach, involving a combination of adequate dietary advice, phosphatebinder use, and adjustments to dialysis prescription.

BMI and MAMC are conventional nutritional assessments for HD patients, and previous studies [38–40] indicated that both are independent predictors of survival. The study conducted by Su et al. [41] exposed that the decrease in MAC was associated with increased all-cause mortality and cardiac events in HD patients, especially in those with low BMI. Unsurprisingly, the data in our study showed that HD patients with decreased BMI and MAMC were more likely to develop sarcopenia, which is consistent with previous studies [42, 43].

Consistent with previous studies [12, 38], This study showed that the level of C-reactive protein was increased in HD patients who developed sarcopenia compared to non-sarcopenia patients. the role of inflammation as a risk factor for malnutrition has been more and more recognized, C-reactive protein is one of the most frequently utilized biochemical indicators to examine inflammation. It has been well established in the field that hemodialysis patients are often under micro inflammatory state for multiple reasons [44]. Indeed, the close association between inflammation and sarcopenia has been well documented. Inflammatory factors can activate numerous signaling pathways involved in the pathogenesis of sarcopenia, resulting in decreased anabolism and increased catabolism of proteins [45]. A systematic review shows that exercise training can be beneficial for both the body composition and nutritional status in hemodialysis patients, the MAMC, BMI, Serum albumin increase and C-reactive protein decrease after resistance exercise [46]. Therefore, attention should be paid to these high-risk patients and early interventions should be taken to improve their outcomes.

Hemodialysis patients often have report significant psychological distress, Depression is common in HD patients [47]. depression patients are significantly less involved in social, professional, and recreational activities resulting in less physical activity [48], Infection [47], these factors are related to the development of sarcopenia. Several previous studies have shown an association between depression and sarcopenia in hemodialysis patients [49–51], there is a higher prevalence of depression in sarcopenia patients. however, we did not find any significant association between depression and sarcopenia. This may be due to differences in study populations and assessment tools.

Currently, commonly used scales for sarcopenia are the SARC-F score and the modified SARC-assisted Cal F score. A meta-analysis [23] revealed that the sensitivity of SARC-F was low to moderate (28.9% – 55.3%), and so was its specificity (68.9% – 88.9%). Although SARC-CalF is associated with higher specificity (87.7% – 91.3%), its sensitivity is not satisfactory (45.9% – 57.2%). The relatively low sensitivity of these two scales renders a higher risk for misdiagnosis. On the contrary, our novel nomogram provided an alternative method with increased clinical efficacy. The AUC of our constructed nomogram model was 0.869 in the development cohort and 0.832 in the validation cohort with an internal validation C-statistic of 0.783. The validity of this novel model was further verified by calibration and DCA curves. The sensitivity, specificity, positive predictive value, negative predictive value, positive likelihood ratio, negative likelihood ratio of our constructed nomogram model were 77%,83%,45%,93%,4.34,0.418, Accuracy was 82%. More importantly, all five variables included in this model are laboratory and anthropometric measurements that are routinely determined in clinical practice and do not require additional examinations or costs. To make this predictive model more convenient for physicians to use in clinical practice, we modified the nomogram into a scoring system with integer points, the scoring system has ease of visualization. If the total score > 121 points indicates that the patient has a high risk of sarcopenia, should be timely comprehensive interventions, such as exercise training, nutritional interventions to reduce the occurrence of sarcopenia.

However, this study has some limitations that need to be taken into account. First, even though the number of enrollments was relatively large, it was conducted at a single center that might not be representative of the HD patients in other areas. Second, our study was retrospectively constructed and hemodialysis patients level of physical activity, serum bicarbonate, vitamin D level and nutritional status were not included in the analysis, potentially reducing the performance of the model. Third, the constructed nomogram model was not validated using external data. Fourth, this study excluded cases with missing data, which may have led to selection bias. Therefore, this model should be validated through prospective, multicenter clinical studies in the future.

## Conclusion

In this study, a predictive nomogram model was constructed based on conventional serology and noninvasive anthropometric measurements that could accurately predict the risk of developing sarcopenia in HD patients. Our model maybe assist physicians in identifying the high risk of sarcopenia in hemodialysis patients earlier and adopt effective intervention strategies to improving patients adverse outcomes. In the future, it is necessary to further optimize the model through prospective, multi-center clinical research.

## Data Availability

The datasets generated and/or analyzed during the current study are not publicly available due to limitations of ethical approval involving the patient data and anonymity but are available from the corresponding author on reasonable request.

## Abbreviations

MHD: Maintenance hemodialysis
ESRD: End-stage renal disease
MRI: Magnetic resonance imaging
CT: Computed tomography
DXA: Dual-energy X-ray absorptiometry
BIA: Bioelectrical impedance analysis
SAS: Self-rating anxiety scale
SDS: Self-rating depression scale
BMI: Body mass index
ASM: Appendicular skeletal muscle mass
ROC: Receiver operating characteristic
AUC: The area under the receiver operating characteristic curve
DCA: Decision curve analysis
Kt/V: Urea clearance index
MAC: Mid-upper arm circumference
MAMC: Mid-upper arm muscle circumference
TSF: Triceps skin fold
PTH: Parathyroid hormone
HDL: High-density lipoprotein
LDL: Low-density lipoprotein
VLDL: Very low-density lipoprotein
SARC-F: Strength, assistance walking, rise from a chair, climb stairs and falls
SARC-CalF: Strength, assistance walking, rise from a chair, climb stairs, falls and calf

## References

[1] Liyanage T, Ninomiya T, Jha V, Neal B, Patrice HM, Okpechi I, Zhao MH, Lv J, Garg AX, Knight J (2015). Worldwide access to treatment for end-stage kidney disease: a systematic review. The Lancet..

[2] Kramer A, Pippias M, Noordzij M, Stel VS, Andrusev AM, Aparicio-Madre MI, Arribas Monzon FE, Asberg A, Barbullushi M, Beltran P (2019). The European Renal Association - European Dialysis and Transplant Association (ERA-EDTA) registry annual report 2016: a summary. Clin Kidney J.

[3] Fahal IH (2014). Uraemic sarcopenia: aetiology and implications. Nephrol Dial Transplant.

[4] Cruz-Jentoft AJ, Baeyens JP, Bauer JM, Boirie Y, Cederholm T, Landi F, Martin FC, Michel JP, Rolland Y, Schneider SM (2010). Sarcopenia: European consensus on definition and diagnosis: Report of the European Working Group on Sarcopenia in Older People. Age Ageing.

[5] Cruz-Jentoft AJ, Sayer AA (2019). Sarcopenia. The Lancet.

[6] Kim JK, Choi SR, Choi MJ, Kim SG, Lee YK, Noh JW, Kim HJ, Song YR (2014). Prevalence of and factors associated with sarcopenia in elderly patients with end-stage renal disease. Clin Nutr.

[7] Mori K, Nishide K, Okuno S, Shoji T, Emoto M, Tsuda A, Nakatani S, Imanishi Y, Ishimura E, Yamakawa T (2019). Impact of diabetes on sarcopenia and mortality in patients undergoing hemodialysis. BMC Nephrol.

[8] Ren H, Gong D, Jia F, Xu B, Liu Z (2016). Sarcopenia in patients undergoing maintenance hemodialysis: incidence rate, risk factors and its effect on survival risk. Ren Fail.

[9] Shafiee G, Keshtkar A, Soltani A, Ahadi Z, Larijani B, Heshmat R (2017). Prevalence of sarcopenia in the world: a systematic review and meta- analysis of general population studies. J Diabetes Metab Disord.

[10] Kim JK, Kim SG, Oh JE, Lee YK, Noh JW, Kim HJ, Song YR (2019). Impact of sarcopenia on long-term mortality and cardiovascular events in patients undergoing hemodialysis. Korean J Intern Med.

[11] Lai S, Muscaritoli M, Andreozzi P, Sgreccia A, De Leo S, Mazzaferro S, Mitterhofer AP, Pasquali M, Protopapa P, Spagnoli A (2019). Sarcopenia and cardiovascular risk indices in patients with chronic kidney disease on conservative and replacement therapy. Nutrition.

[12] Wilkinson TJ, Miksza J, Yates T, Lightfoot CJ, Baker LA, Watson EL, Zaccardi F, Smith AC (2021). Association of sarcopenia with mortality and end-stage renal disease in those with chronic kidney disease: a UK Biobank study. J Cachexia Sarcopenia Muscle.

[13] Ribeiro HS, Neri SGR, Oliveira JS, Bennett PN, Viana JL, Lima RM (2022). Association between sarcopenia and clinical outcomes in chronic kidney disease patients: a systematic review and meta-analysis. Clin Nutr.

[14] Shu X, Lin T, Wang H, Zhao Y, Jiang T, Peng X, Yue J (2022). Diagnosis, prevalence, and mortality of sarcopenia in dialysis patients: a systematic review and meta-analysis. J Cachexia Sarcopenia Muscle.

[15] Cruz-Jentoft AJ, Bahat G, Bauer J, Boirie Y, Bruyere O, Cederholm T, Cooper C, Landi F, Rolland Y, Sayer AA (2019). Sarcopenia: revised European consensus on definition and diagnosis. Age Ageing.

[16] Yoshimura Y, Wakabayashi H, Yamada M, Kim H, Harada A, Arai H (2017). Interventions for Treating Sarcopenia: A Systematic Review and Meta-Analysis of Randomized Controlled Studies. J Am Med Dir Assoc..

[17] Buckinx F, Landi F, Cesari M, Fielding RA, Visser M, Engelke K, Maggi S, Dennison E, Al-Daghri NM, Allepaerts S (2018). Pitfalls in the measurement of muscle mass: a need for a reference standard. J Cachexia Sarcopenia Muscle.

[18] Slee A, McKeaveney C, Adamson G, Davenport A, Farrington K, Fouque D, Kalantar-Zadeh K, Mallett J, Maxwell AP, Mullan R (2020). Estimating the prevalence of muscle wasting, weakness, and sarcopenia in hemodialysis patients. J Ren Nutr.

[19] Malmstrom TK, Morley JE (2013). SARC-F: a simple questionnaire to rapidly diagnose sarcopenia. J Am Med Dir Assoc.

[20] Woo J, Leung J, Morley JE (2014). Validating the SARC-F: a suitable community screening tool for sarcopenia?. J Am Med Dir Assoc.

[21] Lim WS, Chew J, Lim JP, Tay L, Hafizah N, Ding YY (2019). Letter to the editor: case for validated instead of standard cut-offs for SARC-CalF. J Nutr Health Aging.

[22] Duarte PM, Ribeiro HS, Almeida LS, Baiao VM, Inda-Filho A, Avesani CM, Ferreira AP, Lima RM. SARC-F and SARC-CalF are associated with sarcopenia traits in hemodialysis patients. Nutr Clin Pract 2022. 10.1002/ncp.10819 34994475

[23] Voelker SN, Michalopoulos N, Maier AB, Reijnierse EM (2021). Reliability and concurrent validity of the SARC-F and its modified versions: a systematic review and meta-analysis. J Am Med Dir Assoc..

[24] Balachandran VP, Gonen M, Smith JJ, DeMatteo RP (2015). Nomograms in oncology: more than meets the eye. Lancet Oncol.

[25] Dunstan DA, Scott N (2019). Clarification of the cut-off score for Zung's self-rating depression scale. BMC Psychiatry.

[26] Dunstan DA, Scott N (2020). Norms for Zung’s Self-rating Anxiety Scale. BMC Psychiatry.

[27] Chen LK, Woo J, Assantachai P, Auyeung TW, Chou MY, Iijima K, Jang HC, Kang L, Kim M, Kim S (2020). Asian working group for sarcopenia: 2019 consensus update on sarcopenia diagnosis and treatment. J Am Med Dir Assoc.

[28] Fouque D, Kalantar-Zadeh K, Kopple J, Cano N, Chauveau P, Cuppari L, Franch H, Guarnieri G, Ikizler TA, Kaysen G (2008). A proposed nomenclature and diagnostic criteria for protein-energy wasting in acute and chronic kidney disease. Kidney Int.

[29] Kidney Disease: Improving Global Outcomes (KDIGO) CKD-MBD Update Work Group. KDIGO 2017 Clinical Practice Guideline Update for the Diagnosis, Evaluation, Prevention, and Treatment of Chronic Kidney Disease-Mineral and Bone Disorder (CKD-MBD). Kidney Int Suppl. 2017;7:1–59. 10.1016/j.kisu.2017.04.001 PMC6340919 30675420

[30] Yang S, Su T, Huang L, Feng L-H, Liao T (2021). A novel risk-predicted nomogram for sepsis associated-acute kidney injury among critically ill patients. BMC Nephrology..

[31] Cheng Y, Shang J, Liu D, Xiao J, Zhao Z (2020). Development and validation of a predictive model for the progression of diabetic kidney disease to kidney failure. Ren Fail.

[32] Zhu J, Tang C, Ouyang H, Shen H, You T, Hu J (2021). Prediction of all-cause mortality using an echocardiography-based risk score in hemodialysis patients. Cardiorenal Med.

[33] Ouyang H, Shi Q, Zhu J, Shen H, Jiang S, Song K (2021). Nomogram for predicting 1-, 5-, and 10-year survival in hemodialysis (HD) patients: a single center retrospective study. Ren Fail.

[34] Mo YH, Su YD, Dong X, Zhong J, Yang C, Deng WY, Yao XM, Liu BB, Wang XH (2022). Development and validation of a nomogram for predicting sarcopenia in community-dwelling older adults. J Am Med Dir Assoc.

[35] Sanchez-Tocino ML, Miranda-Serrano B, Lopez-Gonzalez A, Villoria-Gonzalez S, Pereira-Garcia M, Gracia-Iguacel C, Gonzalez-Ibarguren I, Ortiz-Arduan A, Mas-Fontao S, Gonzalez-Parra E (2022). Sarcopenia and mortality in older hemodialysis patients. Nutrients.

[36] Palmer SC, Hayen A, Macaskill P, Pellegrini F, Craig JC, Elder GJ, Strippoli GF (2011). Serum levels of phosphorus, parathyroid hormone, and calcium and risks of death and cardiovascular disease in individuals with chronic kidney disease: a systematic review and meta-analysis. JAMA.

[37] Fouque D, Horne R, Cozzolino M, Kalantar-Zadeh K (2014). Balancing nutrition and serum phosphorus in maintenance dialysis. Am J Kidney Dis.

[38] Lin TY, Lim PS, Hung SC (2018). Impact of misclassification of obesity by body mass index on mortality in patients with CKD. Kidney Int Rep.

[39] Noori N, Kopple JD, Kovesdy CP, Feroze U, Sim JJ, Murali SB, Luna A, Gomez M, Luna C, Bross R (2010). Mid-arm muscle circumference and quality of life and survival in maintenance hemodialysis patients. Clin J Am Soc Nephrol.

[40] Stosovic M, Stanojevic M, Simic-Ogrizovic S, Jovanovic D, Djukanovic L (2011). The predictive value of anthropometric parameters on mortality in haemodialysis patients. Nephrol Dial Transplant.

[41] Su CT, Yabes J, Pike F, Weiner DE, Beddhu S, Burrowes JD, Rocco MV, Unruh ML (2013). Changes in anthropometry and mortality in maintenance hemodialysis patients in the HEMO Study. Am J Kidney Dis.

[42] Hortegal EVF, Alves J, Santos EJF, Nunes LCR, Galvao JC, Nunes RF, Lula DA, Carvalho SCR, Franca A, Santos EMD (2020). Sarcopenia and inflammation in patients undergoing hemodialysis. Nutr Hosp.

[43] Lin YL, Liou HH, Wang CH, Lai YH, Kuo CH, Chen SY, Hsu BG (2020). Impact of sarcopenia and its diagnostic criteria on hospitalization and mortality in chronic hemodialysis patients: a 3-year longitudinal study. J Formos Med Assoc.

[44] Carrero JJ, Johansen KL, Lindholm B, Stenvinkel P, Cuppari L, Avesani CM (2016). Screening for muscle wasting and dysfunction in patients with chronic kidney disease. Kidney Int.

[45] Crossland H, Skirrow S, Puthucheary ZA, Constantin-Teodosiu D, Greenhaff PL (2019). The impact of immobilisation and inflammation on the regulation of muscle mass and insulin resistance: different routes to similar end-points. J Physiol.

[46] Bakaloudi DR, Siargkas A, Poulia KA, Dounousi E, Chourdakis M (2020). the effect of exercise on nutritional status and body composition in hemodialysis: a systematic review. Nutrients.

[47] Tian N, Chen N, Li PK (2021). Depression in dialysis. Curr Opin Nephrol Hypertens.

[48] Purnell TS, Auguste P, Crews DC, Lamprea-Montealegre J, Olufade T, Greer R, Ephraim P, Sheu J, Kostecki D, Powe NR (2013). Comparison of life participation activities among adults treated by hemodialysis, peritoneal dialysis, and kidney transplantation: a systematic review. Am J Kidney Dis.

[49] Li Z, Tong X, Ma Y, Bao T, Yue J (2022). Prevalence of depression in patients with sarcopenia and correlation between the two diseases: systematic review and meta-analysis. J Cachexia Sarcopenia Muscle.

[50] Vettoretti S, Caldiroli L, Armelloni S, Ferrari C, Cesari M, Messa P (2019). Sarcopenia is associated with malnutrition but not with systemic inflammation in older persons with advanced CKD. Nutrients.

[51] Yuenyongchaiwat K, Jongritthiporn S, Somsamarn K, Sukkho O, Pairojkittrakul S, Traitanon O (2021). Depression and low physical activity are related to sarcopenia in hemodialysis: a single-center study. PeerJ.

